# The Patent Eligibility of 3D Bioprinting: Towards a New Version of Living Inventions’ Patentability

**DOI:** 10.3390/biom12010124

**Published:** 2022-01-12

**Authors:** Nabeel M. Althabhawi, Zinatul Ashiqin Zainol

**Affiliations:** Faculty of Law, Universiti Kebangsaan Malaysia (UKM), Bangi 43600, Malaysia; shiqin@ukm.edu.my

**Keywords:** 3D bioprinting, products of nature, patentable subject matters, discovery

## Abstract

A combination of 3D printing techniques and synthetic biology, 3D bioprinting is a promising field. It is expected that 3D bioprinting technologies will have applications across an array of fields, spanning biotechnology, medical surgery and the pharmaceutical industry. Nonetheless, the progress of these technologies could be hindered, unless there is adequate and effective protection for related applications. In this article, the authors examine the patent eligibility of 3D bioprinting technologies. This issue raises concern given that existing patent systems are generally averse to nature-derived inventions and many of them exclude products of nature or discoveries from patentability. This qualitative study analyses the current patent systems in key jurisdictions, particularly, the U.S. and the EU, and their applicability, as well as effectiveness, in the context of 3D bioprinting. The study argues that the main reason for the apathy of existing patent systems towards bio-inventions is that they were designed to deal with mechanical inventions. It suggests an innovation framework that encompasses both mechanical and biological inventions to cater adequately to emerging technologies.

## 1. Introduction

The novel technology of 3D bioprinting aims to synthetically produce tissues and other biological constructs by using 3D bioprinters [1]. Presently, this technology has application mainly in the area of medical surgery, where it is used to transplant synthetically printed tissues into patients [2]. Transplanted tissues are usually printed according to a patient’s cells [3]. Sometimes, they are printed according to donors’ cells [4]. According to Bicudo and others, it is hard to pinpoint the precise beginning of bioprinting technologies, although there were some experiments in this area in the middle of the 1980s. It seems that the real emergence of these technologies was in the 1990s, while the vast majority of bioprinting companies were established in the present century [1]. Today, bioprinting is still in an embryonic stage and only simple tissues have been printed. However, future research in this field is expected to enhance the ability to print whole organs and the value of 3D bioprinting will eventually rise to an estimated USD 3 billion in the near future [5]. 

There are three common methods in 3D bioprinting technologies [6,7,8]. First, bioprinting can be by way of the so-called inkjet-based printing. This is a low-cost method. However, it is constrained by the fact that it cannot be used to produce high-viscosity tissues. The second method is micro-extrusion printing, which is a method commonly used to print 3D biological tissues [9]. It overcomes the limitations of the inkjet printing method. Its drawback is that it is a low-resolution form of printing. Third, is the laser-assisted method (LAB), which is less in use than the micro-extrusion technique [10]. However, LAB has some drawbacks, such as the small number of biomaterials that can be transferred, as well as the low speed and high costs, which render LAB uncompetitive in respect to other methods [11].

The first step in 3D bioprinting is the creation of a digital blueprint of the targeted object, that is, a three-dimensional scan of the real object. Where the real object is not available or cannot be scanned, computer-aided design software (CAD) is used to model it [5,12]. The second step is the translation of this blueprint into a path that the printer can follow. This translation is conducted by computer-aided manufacturing software (CAM). The last stage is the printing of the targeted object layer by layer using bioprinters [13]. These bioprinters are robotic devices which function by receiving instructions from software [1]. Nonetheless, it is most significant to understand the microenvironment of the copy tissue before commencing the 3D bioprinting process [5]. 

There are a variety of biomaterials used in 3D bioprinting that can be transplanted from the natural environment without rejection. Hydrogels and sugar are used as a scaffold in the printing process [13]. The scaffold enables 3D-printed cells to replicate [5]. More significantly, bio-ink is a crucial factor in 3D bioprinting technologies. It is essentially made from cells of the same individual (autologous cells) or another individual from the same species (allogeneic cells). Bio-ink can also be made from different species (xenogeneic cells). Furthermore, bio-ink consists of pre-polymer solution hydrogels [9]. 

The 3D bioprinting process faces some problems that are not encountered in conventional 3D printing. For instance, the sensitivity of living cells precludes acceptance by the living organism, that is, the selection of bio-ink components. These problems must be overcome by integrating multiple fields, such as engineering, cell biology, physics and medicine. Until recent years, only a few 3D bioprinting processes have been successful (the result of a patent search conducted by authors on Espatcenet and based on the key phrases “3D bioprinted tissue”, “3D bioprinted organism” and “3D bioprinted organ” showed only 22 issued patents. This is mainly because of some non-natural characteristics, which render the copy organ distinguishable from the original version. For instance, blood vessels, when copied, may create a network of vessels that clearly does not match the network created by the original vessels. Moreover, the thickness of copy tissues must not exceed 200 micros, to allow oxygen to spread between the original organs and the transplanted ones [13].

Technological and biological obstacles apart, 3D bioprinting technologies question the adequacy of the current patent system in protecting inventions arising from this revolutionary field. The issue of patent eligibility has profound implications for emerging technologies as the scene remains unclear [14]. More specifically, patent protection is a key determinant of the progress of 3D bioprinting technologies. With patent protection, more investments and resources would be committed to enhance the state of these technologies. Impliedly, without patent rights, the progress of 3D bioprinting may be hindered [15]. Indeed, the most concerning debate in the patent eligibility of 3D bioprinting applications resonates with the patentability of nature-derived or nature-duplicated breakthroughs and the products of nature doctrine [16].

## 2. Methodology and Materials 

This is a qualitative, theoretical study. It traces law-related articles on bioprinting from the HeinOnline database and LexisNexis. It also involves a discussion of 22 court cases on nature-related patents. Two essential issues are examined. First, the study concisely outlines the potentially debated subject matters of patent protection. As it demonstrates, there is a prospect that some 3D bioprinting patent applications will not face the problem of exclusion suffered by nature-related subject matters. Next, the study delves into the most crucial aspect of the discussion, which concerns nature-related exclusions. The so-called “product of nature” doctrine is discussed under the U.S. patent system, while the discovery exclusion is examined under the European patent system. Lastly, the study suggests that patentable and excluded subject matters should be re-conceptualized so that emerging and potentially valuable technologies are not unduly hindered.

## 3. Results

Many companies prefer patent protection as a proper mechanism to protect bioprinting-related innovations, compared to other types of intellectual property [17]. Here, there are two types of patent protection. The first relates to the bioprinting materials, machines and apparatuses. This type of protection covers bioprinters, bio-ink, scaffold and 3D bioprinting software applications. The second type of protection applies to the processes and products of bioprinting. These include 3D bioprinting process modelling and plantation of bones. It is argued that the patentability of inventions involving 3D bioprinting processes and pre-printing materials, such as bio-ink and scaffold, is more crucial than the outcomes of bioprinting (the bioprinted tissues and organs), because of the limited marketability of these products [5]. Fortunately, the former would be less controversial than the latter in the context of patentability [13]. Moreover, some mechanical inventions, such as printing machines, would not face the controversy over nature-related patents; thus, they would find it easier to receive patent protection, compared to biological inventions, which are more likely to be confronted with the issue of nature-related exclusions [9]. 

### 3.1. The Patentability of Nature-Related Inventions

There has been a debate about the patent eligibility of 3D bioprinting products, whether they are pre-printing materials, printed tissues, or organs, as they are nature-based [14]. Bioprinting products, such as bioprinted tissues and organs, as well as materials used in the printing process, that is, pre-printing materials, would face a challenge regarding their novelty vis-à-vis “natural” biomaterials. Their patentability depends on the level of human ingenuity involved and the extent of their distinction from natural equivalents [9]. Hsiao argues that 3D bioprinting inventions will not pass the eligibility test because their success will depend on the degree to which they resemble naturally occurring ones. It is believed that 3D bioprinting is nothing but a duplication of natural organs, without any “markedly different characteristics” [13]. 

On the other hand, Minssen, Mimler and Boucher contend that bioprinting products are still essentially different from their natural counterparts, thus patent-eligible [9,18]. Xin [19] takes a nuanced position, claiming that perfect 3D bioprinting resembles their original versions, although some of its products may be patent-eligible because they have multiple characteristics that are essentially distinct, such as being constituted by genetically engineered cells. He argues that, although all components of 3D bioprinting are naturally occurring, they may exhibit markedly different characteristics that render 3D bioprinting technologies patent-eligible. He adds that some new qualities can be produced, such as novel and inventive organs. In concluding, he acknowledges that 3D bioprinting products, which are indistinguishable, would not fit within the realm of patent protection based on more recent jurisprudence articulated by the U.S. Supreme Court in cases, such as *Myriad and Roslin*. Nevertheless, Xin proposes some means to have such products patentable. First, he suggests that 3D bioprinting products can be derived from non-natural “intermediate precursors”. Second, products which are a combination of natural and non-natural materials would not be caught by the nature-related exclusions [19]. 

### 3.2. The U.S. “Product of Nature” Doctrine

The U.S. Patent Act provides that patentable subject matters encompass any new and useful process, machine, manufacture, or composition of matter. According to Section 101 thereof, “whoever invents or discovers any new and useful process, machine, manufacture, or composition of matter, or any new and useful improvement thereof, may obtain a patent therefor, subject to the conditions and requirements of this title”. Under the U.S. patent law, eligible subject matters are expansive [20], without any exclusion of patentable inventions, as Congress had intended to protect anything under the sun [21]. Nonetheless, the U.S. courts have espoused that “the scope of the patentable subject matters under that system is broad but it is not endless” [20]. In line with this, current U.S. cases hold, under the so-called “product of nature” doctrine, that phenomena of nature, inter alia, are not patentable.

Sprott asserts that there is no practical guidance as to what constitutes a “product of nature”, nor clarity of its origin [22]. He traced the inception of the “product of nature” doctrine to the U.S. Supreme Court case of *American Wood Paper Co. v. Fiber Disintegrating Co.*, which is believed to be the first case that addressed a nature-related invention. The Court restricted patent protection to the process of extraction, rather than the product itself [23]. Subsequently, it was held by the U.S. Patent Commissioner that discovering a new method to produce a material does not entitle the discoverer to seek patent protection for it [24]. The U.S. Seventh Circuit Court of Appeals endorsed the eligibility of a medicine purified with a useful difference, which makes the compound “therapeutically available” [25]. This case adopted the “therapeutic value test”, which bases patent eligibility, inter alia, on medical utility [22]. 

The therapeutic value test was cited by judge Learned Hand in the case of *Parke-Davis & Co. v. H.K. Mulford Co.*, where he applied the purification rule to a biologically purified adrenaline that was lifted out from its natural environment to produce “a new thing commercially and therapeutically” [26]. The therapeutic value test was reaffirmed by the Seventh Circuit in *Dennis v. Pitner*. The plaintiff brought this case alleging infringement of a patent issued to protect an insecticide that was extracted from the roots of the cube plant. In reply, the defendant argued that the patent was incompatible with the law, as the purported invention was a mere revelation of nature. However, the Court rejected this proposition, relying on the patent’s “value to mankind”. Remarkably, the Court stressed that the application of the “laws of nature” principle has been confusing and devoid of clarity [27]. 

The therapeutic value test survived, even after the enactment of the U.S. Patent Act of 1952. The District Court of Columbia had an opportunity to examine the patentability of a purified arterenol compound, which the Commissioner of Patent had declared patent-ineligible. After close examination of the matter, the Court concluded that the rejection ignored the fact that isolation and purification are necessary to attain therapeutic value [28]. Subsequently, it was clarified, in *Merck & Co. v. Olin Mathieson Chemical Corporation*, that the U.S. Patent Act does not “preclude the issuance of a patent upon a “product of nature”, when it is a “new and useful composition of matter” and there is compliance with the specified conditions for patentability. All the tangible things with which man deals and for which patent protection is granted are products of nature in the sense that nature provides the basic source materials” [29].

The patent eligibility of materials isolated from their natural environment was again on the table of discussion in the U.S. Court of Customs and Patent Appeals (CCPA) in *Application of Bergstrom* [30]. The case came to the Court after the Patent Office rejected an application to patent two compounds that had the effect of simulating smooth muscles and decrease blood pressure. The justification for the rejection by the Patent Office was the lack of new properties, compared with the non-purified forms of the compounds. Taking issues with the attorney’s position, which deemed the compound as “naturally occurring”, the Court concluded that the claimed compounds did not exist. It held that the pure materials were “new” within the meaning of the statutory requirement of novelty [30]. 

The patentability of isolated materials has proved controversial. As Sprott argues, the patentability of isolated and purified substances was a significant loophole in the “product of nature” doctrine. Multiple patents were issued, despite their nature-originated claims [22]. The problems associated with isolation and purification became more perplexing with advancements and breakthroughs in the field of biotechnology. The borderlines between products of nature and products of man became increasingly vague. This strengthened the position of those opposed to the exclusion of products of nature. The inclusion of “living products” within the meaning of statutory patentable subject matters was first initiated by the U.S. Court of Customs and Patent Appeals in *Application of Bergy* [31]. The Court held that there was no ground in Section 101 of the U.S. Patent act, even if strictly construed, to exclude a manufacture or composition of matter because it was alive. 

Notwithstanding, there can be no satisfactory discussion on the “product of nature” doctrine without referring to the landmark case of *Diamond v. Chakrabarty* [21]. In *Chakrabarty,* the U.S. Supreme Court parted with the general understanding that living microorganisms could not be patented and opened the gate to the patenting of many genetically modified living organisms [27]. The Court referred to the wide interpretation of the term “manufacture”, which was given, in *American Fruit Growers, Inc. v. Brogdex Co*. [32], as “the production of articles for use from raw or prepared materials by giving to these materials new forms, qualities, properties, or combinations, whether by hand-labour or by machinery”. 

Hsiao points out that a two-phase test was conducted by the Court in *Chakrabarty* to render the invention a “product of man”. First, the invention shall be the result of human ingenuity. Second, the invention shall bear different characteristics from its natural counterparts [13]. Agarwal and Agarwal argue that 3D bioprinting inventions have a good prospect of passing the second prong of the *Chakrabarty* test. As they explain, there are substantial differences between 3D bioprinting products and their natural counterparts. First, some essential features, such as innervation, are not printable. Second, the aggregation of printed cells is not analogous to the aggregation of natural ones. Third, some 3D bioprinting products are the results of the combination of natural and artificial materials [16]. 

After *Chakrabarty,* many patents were granted for modified and isolated genes. Approximately, 47,000 of such patents were issued [33]. However, the debate led US Patents and Trademark Office (USPTO) to elucidate the foggy scene in 1989 by stating that it would consider “non-naturally occurring, non-human multicellular living organisms, including animal”. The position taken in *Chakrabarty* was reaffirmed in the leading case of *Amgen Inc. v. Chugai Pharmaceutical Co., Ltd.* [34]. In *Amgen,* the U.S. Court of Appeals for the Federal Circuit upheld the decision of the District Court of Massachusetts, which found that claims directed to a purified and isolated DNA sequence were patentable. According to Sprott, *Amgen* paved the way to the filing of many applications of purified and isolated DNA [22]. However, the USPTO, in a 1989 statement, excluded claims directed to or including human beings from the above rule. Cho [33] connects the USPTO’s statement with the Leahy–Smith America Invents Act (AIA) of 2011, which states that “no patent may issue on a claim directed to or encompassing a human organism”. However, Ebrahim points out that, whereas the terms “directed to” and “encompassing” are well-known, the phrase “human organism” is undefined; understanding of it is lacking in both the U.S. courts and the USPTO [14]. 

The turning point in the modern history of the patentability of genetic inventions came in 2012, when the U.S. Supreme Court heard the case of *Mayo Collaborative v. Prometheus Labs.* Discussing a patent directed to a process aimed at assisting medical personnel to use thiopurine drugs to confront autoimmune diseases by the determination of the dosage level, the Court concluded that the process had not transformed the laws of nature into a patent-eligible application [35]. However, it did not reject the inclusion of laws of nature in an invention, if it contained what the Court called an “inventive concept”. The long story about the patentability of genes came to its end in 2013, when the U.S. Supreme Court decided the renowned case of *Ass’n for Molecular Pathology v. Myriad*. In *Myriad*, the Court held that isolated genomic DNAs are “products of nature” as they identical to naturally occurring ones, without any human ingenuity which should have intervened to create or alter the DNAs. The mere isolation of genes does not qualify for patent protection [36]. 

In 2014, the USPTO issued an interim guidance for the determination of the patent eligibility of nature-derived inventions. The guidance provides that, first, to test the patentability of an invention, the claim must relate to a process, manufacture, machine or composition of matter. The guidance then proceeds to the “judicially recognized exceptions” test, which has two prongs. In the first prong (2A), it shall be determined whether the claim is directed to a “product of nature” or a natural phenomenon. If so, the second prong (2B) shall be conducted to identify any potential additional elements that vest the claim with “markedly different characteristics” [37]. 

**Figure 1:**
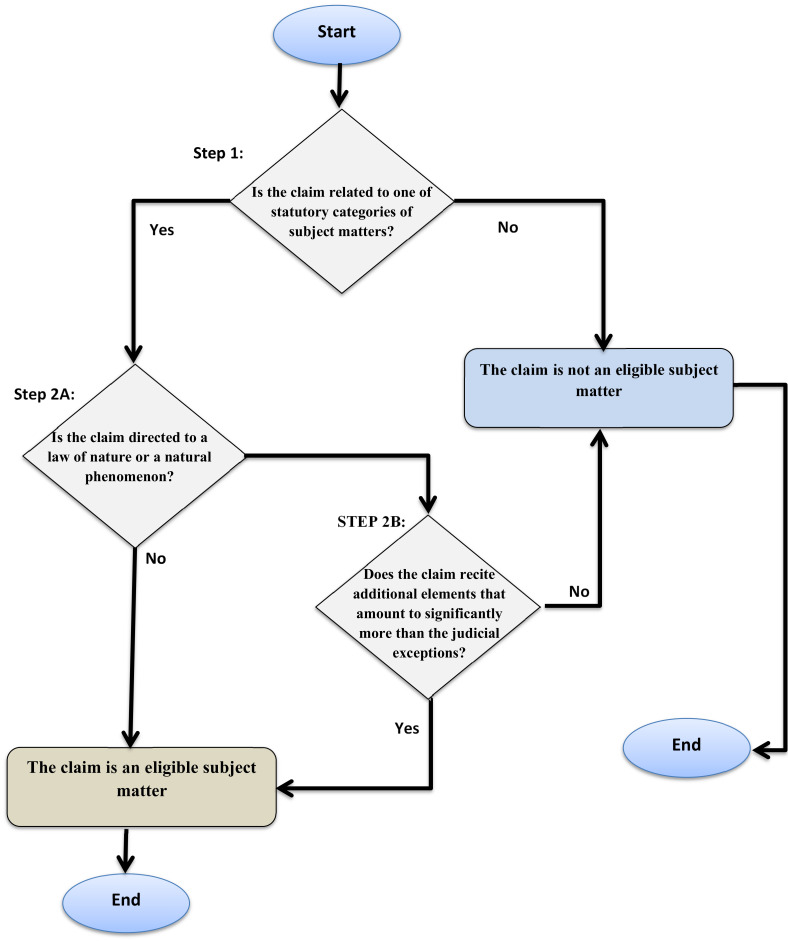
The USPTO’s nature exclusion test.

The Court of Appeals for the Federal Circuit held, in the 2014 case of *Roslin*, that it was not correct to argue that the copies (clones) of a sheep were patentable as the results of human ingenuity. The copy, according to the Court, was “an exact genetic replica of another sheep” and did not possess “markedly different characteristics from any [farm animals] found in nature” [38]. The Court rejected patent protection for environment-generated characteristics, insisting that patentability shall only be extended to protect human ingenuity. In response to the decision in *Roslin*, the USPTO released the Revised Patent Subject Matter Eligibility Guidelines (RPEG) [39]. The central point of the guidelines is the case law-created exclusions. The guidelines elaborate step 2A in the interim guidance, which excludes claims “directed to a judicial exception”. The RPEG sets two prongs for that test. First, if an abstract idea, a law of nature or natural phenomenon is recited in a claim, then, according to the RPEG, the mere inclusion is a recitation. If there is a recitation, then a patent examiner must proceed to the second prong of the test. The question, here, would be whether there is any practical application that lifts a claim out of the exclusions (see Figure 1)

On the other hand, a congressional draft bill was released in May 2019. The draft stated that “no implicit or other judicially created exceptions to subject matter eligibility, including “abstract ideas”, “laws of nature”, or “natural phenomena”, shall be used to determine patent eligibility under Section 101 and all cases establishing or interpreting those exceptions to eligibility were thereby abrogated. The eligibility of a claimed invention under Section 101 shall be determined without regard to the manner in which the claimed invention was made; whether individual limitations of a claim are well known, conventional or routine; the state of the art at the time of the invention; or any other considerations relating to Sections 102, 103, or 112”.

### 3.3. The EU Exclusion of Discovery 

In Europe, the issue of nature-derived patents is less complicated. This is because statutory exclusions exist that put some breakthroughs out of the patent realm. While, in the U.S., the issue of exclusions is two-fold—what is the law and how it is applied—the debate, in Europe, relates only to the second fold. Article 52(2) of European Patent Convention (EPC) provides that “(2) the following, in particular, shall not be regarded as inventions within the meaning of paragraph 1: (a) discoveries, scientific theories and mathematical methods…” Moreover, Article 53 of this instrument excludes “(b) plant or animal varieties or essentially biological processes for the production of plants or animals—this provision shall not apply to microbiological processes or the products thereof; and (c) methods for the treatment of the human or animal body by surgery or therapy and diagnostic methods practiced on the human or animal body—this provision shall not apply to products, in particular substances or compositions, for use in any of these methods.”

Nevertheless, patent applications, which consist of any excluded subject matters, would be, theoretically, patent-eligible, if they introduce an industrial application based on the exclusion. In *Aerotel v. Telco Holdings*, the UK Court of Appeal held that “[a] physical embodiment, such as a cloning vector employing knowledge of the discovery of a DNA sequence is not discovery as such” [40]. The implementation regulations of the EPC require the invention to have technical features related to a technical field that are a concern to a technical problem. On this basis, it has been argued that elements of technicality are present in 3D bioprinting, thus rendering it patentable [9]. 

In 1998, the European Biotechnology Directive was adopted. This Directive is one of the most significant milestones in the history of the European patent system, as it legislatively introduced new principles, which had proved controversial in the pre-1998 European jurisdiction. The European Patent Office (EPO) relies heavily on this Directive in the context of biotechnology inventions, generally, and isolated genes, in particular [41]. Lindhorst argues that the language of this Directive is analogous to the one that was used by the U.S. Supreme Court in *Chakrabarty* [42]. 

The second chapter of the Directive provides constructions of the patentability criteria set by the EPC. In Article 3, the Directive states the following: “2. Biological material which is isolated from its natural environment or produced by means of a technical process may be the subject of an invention even if it previously occurred in nature”. Article 3(1) of the Directive excludes plant and animal varieties and essentially biological processes to produce plants or animals from patentability. Article 3(2) provides a possibility for the patenting of an invention relating to plants and animals, if “the technical feasibility of the invention is not confined to a particular plant or animal variety”. According to Article 3(3), the application of the exclusion of essentially biological processes to produce plants or animals “shall be without prejudice to the patentability of inventions which concern a microbiological or another technical process, or a product obtained by means of such a process”.

Regarding gene patents, the Directive clarifies that an invention directed to an element isolated from the human body is patent-eligible. Unlike the U.S. patent system, the Directive extends patent protection to isolated elements that are identical to their natural counterparts [43]. The Directive justifies the protection of these inventions as the outcomes of a technical process that put on the ground what the human body does not. Following a parliamentary vote against the adoption of this Directive, The Netherlands brought an action for its annulment before the Court of Justice of the European Union (CJEU) under Article 230 of the EC Treaty. The CJEU refused the request for annulment. In its decision, the Court set out two elements, which any claim seeking such patents must demonstrate. These are “a description of the original method of sequencing which led to the invention and an explanation of the industrial application to which the work is to lead, as required by Article 5(3) of the Directive. In the absence of an application in this form, there would be no invention, but rather the discovery of a DNA sequence, which would not be patentable as such” [44]. 

However, the controversy over the patentability of biotechnological inventions in the EU is largely ethically oriented [45]. This is reflected in the German case of *Oliver Brüstle v. Greenpeace* (2011) [46] and the UK case of *International Stem Cell Corporation v. Comptroller-General Of Patents, Designs And Trade Marks* (2015) [47]. Regarding the discovery exclusion, it is opined that the borderline between invention and discovery lies in the requirement of industrial property, which illustrates the human contribution that may qualify for patent protection [48]. In conclusion, the case law practice in the EU illustrates the complications surrounding the application of patent law to biotechnological inventions. Judicial espousal recurrently construes the statute law in different ways according to scientific developments. This renders judicial interpretation vital in determining patentable subject matters [45].

## 4. Discussion

It can be asserted that the patent system has been struggling to cope with every emergence of a new, revolutionary technology. This is the result of multiple, divergent factors that shape the patent protection landscape. Patents are mainly designed to protect non-routine scientific findings that are useful. The starting point of this protection is the identification of the subject matter sought to be protected. However, this task has not been straightforward. Two types of complications are associated with it. First, there is a conflict between law as a field that relies much on stability, on the one hand, and the scientific disciplines, which are based on fast game-changer breakthroughs, on the other. Second, the concept of invention is required to apply across all fields whose findings are protected by patents. Moreover, this applicability is required to be equal across all fields. This obstacle affects, inter alia, the determination of subject matters. For instance, Burk and Lemley observe that there has been a growing divergence between patent law rules and their application across the spectrum of technologies [49]. Ebrahim elaborates that, with regard to subject matters, there is no uniform treatment of technologies. There have been many subjective interpretations by the courts as to what merits patent protection. These different and sometimes contradictory interpretations result in inconsistencies in case-law rules. Ebrahim stresses that this recurrent change in judicial espousal may render vague and uncertain the patent eligibility criteria in the context of 3D bioprinting [14]. 

The development of a uniform conception of patentable subject matters is not just hindered by the existence of vastly different scientific disciplines. It is also affected by the dominance of some particular fields over others. This study can assert that the conventional patent system was not designed to deal with biotechnology. At the time when the current patent systems were designed, mechanical inventions were overwhelmingly dominant. This fact was argued by the Commissioner of the USPTO in *Chakrabarty* [21]. While it is difficult to accept the USPTO Commissioner’s conclusion, that legislator-unforeseen technologies necessitate legislative intervention, it is right to admit that biotechnology and nature-derived inventions were not contemplated when the modern patent systems were fashioned. 

To cope with the challenges that have followed biotechnological inventions, many changes to the rules of patentable subject matters have occurred. The aim of these changes was to identify patentable subject matters by defining “invention”. However, every attempt to do so has added mystery and vagueness to the concept, making a precise definition a seemingly unachievable goal. Despite repeated attempts made in numerous cases, as well as in the literature, to establish a definition, no acceptable definition has been found that can apply across different scientific fields. The term “invention” has had several ambiguous definitions for almost a century of judicial practice. For instance, the term has been defined variously as “an exercise of the inventive faculty” [21], “an exercise of inventive skill” [50], “the creative work in the inventive faculty” [51], “substantial invention or discovery” [52] and “the flash of creative genius” [53]. It has even been asserted that “invention” is a term that is undefined and cannot be defined precisely. Thus, the judgment as to whether or not a case involves an invention is subjective and inconstant. According to judge Learned Hand, “[a]n invention is a new display of ingenuity beyond the compass of the routines, and in the end, this is all that can be said about it… We must try to correct our standard by such objective references as we can, but in the end the judgment will appear, and no doubt be, to a large extent personal, and in that sense arbitrary” [54]. 

This study makes two observations regarding efforts aimed at determining patentable subject matters. First, all definitions seem to be directed to the act of inventing, rather than the outcome that is to be protected at the end of the day. It is conceivable that an invention can be the result of a routine activity and vice versa. Hence, attention should be directed to the protected subject matter. For instance, Percy LeBaron Spencer invented the microwave oven accidently in the course of a routine job [55]. Second, it should be borne in mind that what should be identified, in the context of patentable subject matters, is the concept of invention, rather than the patentable invention. The latter can be determined by looking out for the presence of the three patentability criteria, which are novelty, inventive step (non-obviousness) and industrial applicability (utility).

Cho points out that such a distinction was absent in some cases in which the courts discussed the subject matter in the light of other prerequisites, such as novelty and non-obviousness. According to him, in *Merck,* the court discussed the “product of nature” in the novelty and non-obviousness contexts, which resulted in the invalidation of the patent at issue [33]. More significantly, Osenga contends that the USPTO’s allusion to intensification in the debate about protectable subject matters could be explained by the fact that the courts were discussing the issues of novelty and non-obviousness in the context of Section 101 of the U.S. Patent Act [56]. In 1979, the U.S. Court of Customs and Patent Appeals opened the door to the distinction between inventions and patentable inventions. According to the Court, some inventions are not patentable, even though they are deemed as inventions [31]. 

Consequently, the right path to addressing the problem of patentable subject matters is to rely solely on the concept of invention to identify the relevant subject matter, while keeping in mind that the three patentability requirements would preclude routine inventions that can be made by a person skilled in the art from entering the patent realm. Many human activities involve a degree of inventiveness, so that “the term invention embraces all new developments in the social, administrative, business, technical, scientific, and aesthetic fields” [57]. The extensive meaning of subject matters can be narrowed down by the three-legged stool of patentability. Any attempt to differentiate between mere routine and high-level inventions and to use the latter as a yardstick for identifying patent-eligible inventions would likely be outstripped by the pace of scientific progress [58]. 

This study argues that the proper way to avoid the overlap between subject matters and the three patentability requirements is to adopt a broad concept of invention by merely adhering to its literal meaning. Lexically, invention means “[t]he original contrivance or production of a new method or means of doing something, of an art, kind of instrument, etc. previously unknown; origination, introduction” [59]. In this sense, any new finding can be deemed as an invention. The scope of protection can then be restricted by using the three criteria for patentability. This broad language is evident in the U.S. Patent Act, which starts its conceptualization of subject matters by using the term “any”. The U.S. Supreme Court construed this wording as having been intended to provide a wide scope for subject matters [21]. Similarly, the EPC used the same term to broaden the scope for patent protection. Undoubtedly, this conception would cover 3D bioprinting inventions. This is because they are new methods and productions, which do not fall short of the meaning of invention.

Ammar argues that the broad definition of manufacturing and composition of matter adopted by the U.S. Supreme Court can help to incorporate 3D bioprinting products within this extended meaning of invention and render them patent-eligible [5]. The Court defined composition of matter as “all compositions of two or more substances and includes all composite articles, whether they be results of chemical union, or of mechanical mixture, or whether they be gases, fluids, powders or solids” [60]. 

**Figure 2:**
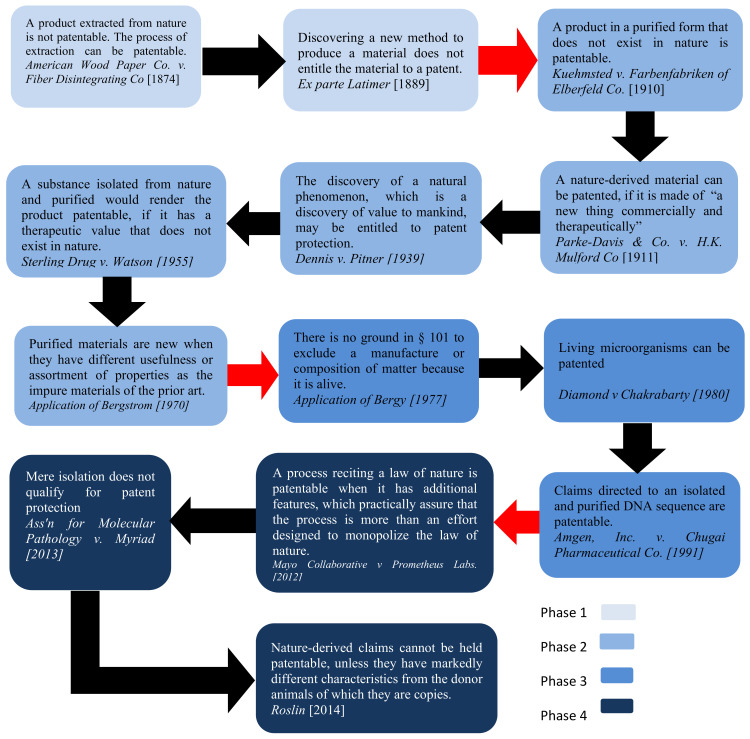
Evolvement of the assessment of nature-derived inventions in U.S. Patent System. Note: the red arrows demonstrate the transformation from phase to phase.

The second problem of even greater concern regarding the apathy towards the patentability of nature-related inventions such as 3D bioprinting is the laws of nature or discovery exclusion, which is not discussed in this study. Here, one should underline the evolvement of the patent–nature relationship. As aforementioned, the patent system was not, in its early application, designed to cover nature-based inventions. Figure 2 below illustrates four phases of the evolvement of the assessment of nature-derived inventions under the U.S. patent system. In the first phase, the courts, when confronted with a new advancement in extracting matters from a natural environment, tended to confine protection to the human-made aspect of the invention, which can be referred to as the process of “disassembling nature”.

In the second phase of that evolution, which started with *Kuehmsted* and *Parke-Davis,* protection was extended to cover purified materials, which did not exist in nature. Progress in the scientific fields pushed inventive activities from simple extraction to more sophisticated isolation and purification of entirely natural matters. The courts then relied on the “therapeutic value test”, which can be related to the utility requirement, rather than the applicability of the concept of inventions to newcomers. This marked another mix-up between patentable subject matters and the patentability criteria. The “purifying and isolating nature” phase was not retarded by the application of the inconstant case-law. Many patents were issued, regardless of their nature-originated claims [22]. Until the 1970s, nature-derived products were non-living. However, in *Bergy* and *Diamond*, a complication was added with the emergence of the notion of living subject matters. This could be seen as the third phase of the evolution process. In this phase, patent applications started to incorporate claims bearing terms such as “manipulating and modifying nature”.

This put the spotlight on the manipulation and modification of life and added more fuel to the controversy over nature-related inventions. With the increasing convergence of biology and technology, the current state of patentability has moved to the most perplexing part of the story. Biotechnology findings have rendered conceivable breakthroughs possible. The debate now is whether “creating life” outcomes is patentable. In the patent context, the new field of synthetic biology marks the differentiation between nature and life. Both of these notions had previously been seen as interchangeable. The separation of the two concepts is vital to the resolution of the problem concerning the mystery of the patentability of living organisms. Non-natural living organisms signal the beginning of a new era in the patent system. Adherence to the conventional patentable subject matters is no longer tenable in light of today’s technological revolutions. Biomaterials are playing an increasingly vital role in numerous industrial sectors [61]. Arguably, 3D bioprinting provides a meeting point between biomaterials production and industries, as it enables the manufacture of multiple copies of one design. Additionally, bio-electronic would benefit from 3D bioprinting technologies [62]. It is conceivable that future industries will rely more on devices and machines made entirely of living components. Indeed, it would not be going too far to assert that the boundaries between living and non-living things are eroding with the advent of 3D bioprinting [14].

As mentioned already, the patent eligibility of 3D bioprinting products will be one of the key factors that will enhance research and development in this field. There is an increasing number of arguments to sustain the applicability of the patent system to 3D bioprinting inventions. For instance, Ammar argues that the fact that 3D bioprinting inventions incorporate a “biologically active organism” does not negate their patentability when the other patentability requirements are met [5]. In addition, Ebrahim insists that the notion of “markedly different characteristics” needs to be revisited and properly defined in the context of 3D bioprinting [14].

Surely, those arguments have merit, but more comprehensive reforms are needed. The modern patent law needs a reconceptualization of eligible and excluded subject matters. As mentioned earlier, patentable subject matters should include every man’s creation. The concern that routine and non-inventive works might be patented will not be eliminated by the exclusions. The other three requirements for patentability are adequate to exclude a nature-based invention from protection on the grounds that it is pre-empted by the prior art or lacks an inventive step or industrial applicability. This does not render the mere revealing of natural facts patentable. A patentable subject matter must be the result of human ingenuity.

The boundaries of discovery or products of nature should be redrawn. Nature and life are no longer concomitant. Moreover, natural materials may be components of patent-eligible inventions. The nature of the components in claimed inventions should not be a barrier to their patentability, which should ideally depend more on the new way in which those components have been combined [63]. It is very relevant, here, to quote judge Markey, who, in discussing the controversy over the patentability of combinations, stated that “[o]nly God works from nothing. Man must work with old elements” [64].

## 5. Conclusions

The authors of this article make two suggestions to cope with 3D bioprinting inventions in the context of the law of nature exclusions. First, attention should be paid to a broad interpretation of invention, instead of focusing on a high level of inventiveness. This would prevent an overlap between the issue of patentable subject matters and the patentability requirements of novelty, inventiveness and industrial applicability. Second, there is a need to redraw the boundary of exclusions by differentiating between nature and life. This is because nature is no longer the exclusive creator of living organisms. It is the authors’ prediction that biomaterials will be the most essential drivers of future industries.

## Data Availability

Not applicable.
